# The Micro-Shear Bond Strength of Various Resinous Restorative Materials to Aged Biodentine

**DOI:** 10.22037/iej.v13i3.20880

**Published:** 2018

**Authors:** Mohammad Hossein Nekoofar, Fariba Motevasselian, Mansoreh Mirzaei, Esmaeil Yassini, Hoda Pouyanfar, Paul MH Dummer

**Affiliations:** a *Department of Endodontics, Dental School, Tehran University of Medical Sciences, Tehran, Iran; *; b *Endodontology* *Research Group, Dental School, College of Biomedical and Life Sciences, Cardiff University, Cardiff, UK; *; c *Department of Restorative and Esthetic Dentistry, Dental School, Tehran University of Medical Sciences, Tehran, Iran*

**Keywords:** Bond Strength, Composite Resin, Dental Adhesive, Glass Ionomer Cement, Tricalcium Silicate

## Abstract

**Introduction:**

The type of materials and application time of veneering restorations on calcium silicate cements are important factors which influence the interfacial properties. The aim of this study was to measure the micro-shear bond strength of a resin composite (RC) using several adhesive systems and a resin-modified glass ionomer cement (RM-GIC) to different aged Biodentine specimens.

**Methods and Materials:**

A total of 15 Biodentine blocks were prepared and assigned to three aging periods: 12 min, one week and one month. Then they were subdivided into five sub-groups to receive cylinders of resinous materials. RC was applied using different adhesive systems: A) no adhesive B) etch and rinse C) two-step self-etch and D) universal adhesive in self-etch mode and E) RM-GIC applied directly over Biodentine. Micro-shear bond strength was measured and the data were analyzed using one-way and two-way ANOVA. The level of significance was set at 0.05.

**Result:**

There was significant interaction between Biodentine aging periods and resinous materials (*P<*0.05). The highest value was obtained in group D bonded to the recently set Biodentine. Increasing the aging period to one week resulted in increased micro-shear bond strength in all groups expect for group D. One-month incubation time led to reduced shear bond strength in group A, C and D. Micro-shear bond strength values of group E increased to the longer aged Biodentine.

**Conclusion:**

Group D showed the highest bond strength to freshly mixed Biodentine.

## Introduction

Over the last decade, mineral trioxide aggregate (MTA) has gained great popularity as a pulp capping agent because it has proper physical and regenerative characteristics [1], has low solubility after setting [2], can set in a wet environment, and induce mineralized tissue formation [3]. Despite its unique properties, MTA has several shortcomings, namely its prolonged setting time [4], its high solubility during setting [5], its potential for discoloration [6] and its difficulty in handling [5]. In addition, the hydrophilic nature of the material and its delayed maturation complicate the application of the final restorative material at the same visit, which usually necessitates scheduling a second appointment after MTA has reached its initial set [7]. The etching and rinsing procedures required for adhesive restorative materials may dislodge unset MTA [8, 9] and adversely affect its physical properties [10, 11].

With the introduction of alternative calcium silicate-based material such as Biodentine (Septodont, Saint-Maur-des Fosses, France) there is an opportunity for the clinical procedure to be simplified because of its combined capability of vital pulp treatment and short setting time [9, 12]. According to the manufacturers’ instruction the setting time of Biodentine is 12 min [13] and it has been suggested that resin composites and GICs can be placed over set Biodentine immediately after this time [14, 15], which would enable single-visit procedures.

The durability of the adhesive bond between Biodentine and the resin composite is of clinical significance with regards to the longevity and predictability of the veneering material [9, 14, 16]. The bond durability also may be affected by the type of adhesive system, self-etch and/or etch and rinse adhesives [16].

The aim of this laboratory study was to determine the micro-shear bond strength of a resin composite using three different adhesive systems (a total etch and rinse adhesive, a two-step self-etch adhesive and a universal bonding agent in self-etch mode) or a resin modified glass ionomer cement (RM-GIC) to Biodentine aged over time. It was hypothesized that there is no difference in the micro-shear bond strength within various resinous materials bonded to different aged Biodentine blocks.

## Materials and Methods

A total of 15 acrylic rectangular molds (20×7×2mm) were prepared and placed on wet pieces of gauzes. The acrylic molds were fully filled with mixed Biodentine and it was gently packed using a smooth surface condenser with minimal pressure. 

Then, a glass slab was gently dragged across the top surface of the mold, rather than placed on Biodentine, to create a flat, smooth surface for later application of cylinders of resin composite or glass ionomer over a relatively standardized smooth surfaces among 15 blocks. All the blocks were then stored at 37^°^C in a chamber to provide fully saturated humidity (PECO Model 455G, Pooya Electronic Co., Tehran, Iran) for different aging periods: 12 min, one week and one month. After each period of incubation, the blocks were randomly selected and divided into five groups to receive very small cylinders, eight each, of the following resinous materials:

Group 1-RC with no adhesive application (negative control group); group 2-RC with etch-and-rinse adhesive system (Adper^TM ^Single Bond 2,3M/ESPE, St.Paul, MN, USA); group 3-RC with two-step self-etch adhesive system (Clearfil SE Bond, Kuraray, Okayama, Japan); group 4-RC with universal dental adhesive in self-etch mode (All-Bond Universal, Bisco, Schaumburg, IL, USA); 5-RMGI (Fuji II LC, GC, Tokyo, Japan). 

**Table1:** List of materials used in the current study

**Material**	**Composition**	**Steps for application**
**Biodentine **	Powder: Tri-calcium silicate, Di-calcium silicate, Calcium carbonate and oxide, Iron oxide, zirconium oxide Liquid: Calcium chloride, Hydro soluble polymer	One dose of liquid and powder mixed for 30 sec at 4000 rpm in an amalgamator
**Composite **	Bis-GMA, UDMA, TEGDMA, Bis-EMA, PEGDMA, silica filer, zirconia filler	Light cure for 20 sec
**Adper Single Bond 2 **	BISGMA, HEMA, dimethacrylates, a methacrylate functional copolymer of polyacrylic and polyitaconic acids, ethanol, water, silica fillers, Camphorquinone	Apply 37% phosphoric acid etchant for 15 sec Rinse for 10 sec Apply 2 or 3 consecutive coats of adhesive Allow gentle air stream for 5 seconds Light cure for 10 sec.
**Clearfil SE Bond **	Primer: MDP, HEMA, DMA ,water Bonding: MDP, HEMA, DMA, Bis-GMA, Filler, Camphorquinone.	Apply primer for 20 sec. Dry with mild air flow Apply bond and distribute evenly with mild air flow Light cure for 10 sec
**All-Bond Universal**	HEMA, BISGMA, MDP, water, ethanol, initiator, Accelerator	*1.* Apply two separate coats *2.* Evaporate excess solvent by air-drying with an air syringe for 10 sec *3.* Light cure for 10 sec
**Resin modified glass ionomer **	Powder: Fluoro-alumino-silicate glass Liquid: Polyacrylic acid, HEMA, Distilled water, Urethane dimethylacrylate, initiator Camphorquinone	Place one scoop of powder and two drops of liquid on pad. Pull half of the powder onto liquid and mix with lapping strokes for 10 to 15 sec Pull in remaining powder and mix thoroughly not exceeding 25 sec Light cure for 20 sec

**Figure 1:**
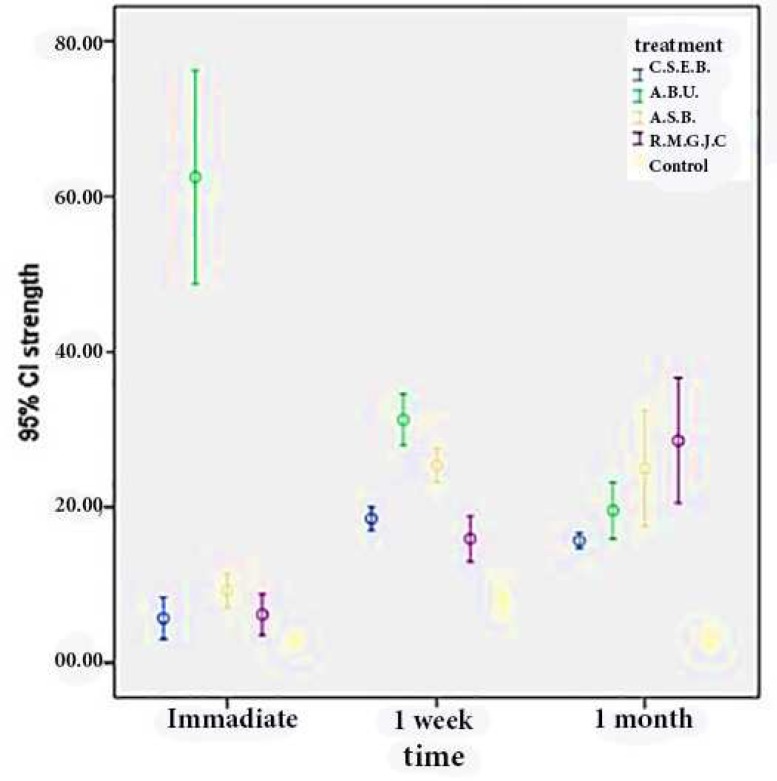
Mean micro-shear bond strength and the 95% confidence intervals of different resinous materials to aged Biodentine^TM ^(C.S.E.B: Clearfil SE Bond, A.B.U: All-Bond Universal, A.S.B: Adper^TM ^Single Bond 2, R.M.G.I.C: Resin modified glass ionomer cement)

The adhesive bonding agents were light cured for 10 sec and silicone matrices with an internal diameter of 0.7 mm and a height of 1 mm were positioned on top surface of each block and filled with resin composite and glass ionomer and cured with a light-emitting diode light-curing unit (Demetron LC, SDS Kerr; Orange, CA, USA) with an intensity of 400 mW/cm^2^ for 20 sec.

The plastic matrices were then removed from the composite (Filtek Z350 XT, 3M/ESPE, St. Paul, USA) and RM-GIC cylinders using a razor blade. All the blocks were maintained for two days at 37^º^C at a relative humidity of 100% before the micro-shear bond strength test.

The manufacturers, the stages for application of the materials and their chemical composition are summarized in Table 1.

***Micro-shear bond strength***

The micro-shear bond strength test was performed using the bond tester machine (Bisco, Inc., Schaumburg, IL, USA). The Biodentine^TM ^blocks were placed into the apparatus with a cyanoacrylate adhesive. A thin wire with a diameter of 0.2 mm was looped around the resin composite or RM-GIC cylinders. A shear force was applied to each specimen at a cross-head speed of 0.5 mm/min until fracture. The micro-shear bond strength was calculated in MPa by dividing the peak load at failure (N) over the surface area (mm^2^) of the specimen.

***Statistical analysis***

All data were analyzed using SPSS version 18 (SPSS, Inc., Chicago, IL, USA). Normal distribution of data was confirmed using one sample Kolmogrov-Smirnov (*P*>0.05). As a result, Variance Analysis was used to analyze the data. The effect of various incubation periods and adhesive materials on bond strength was compared using two-way ANOVA. Since the interaction was significant, one-way ANOVA analysis and Tamhane test (Levin’s test *P*<0.05) were used for statistical comparison of subgroups with Bonferroni adjustment. The level of significance was set at 0.05. 

## Results

The mean values and standard deviations of micro-shear bond strength and the comparison between all the experimental groups have been presented in Table 2 and Figure 1.

The bond strength values were influenced by the Biodentine aging periods*.*

Among different adhesive systems, All-Bond Universal revealed the highest micro-shear bond strength to 12-min aged Biodentine blocks (62.44±16.39 MPa) and control group showed the lowest value (2.76±0.62 MPa).

Increasing the aging period of Biodentine specimens to one week significantly resulted in increased bond strength values at all adhesive groups (*P*<0.001) in comparison to 12 min except for All-Bond Universal which significantly decreased (*P*=0.003). One-month aging period of Biodentine block resulted in significantly lower bond strength values at control, Clearfil SE Bond and All-bond Universal groups in comparison to one-week incubation time (*P*<0.01). No significant difference was observed between one week and one month micro-shear bond strength values at Adper Single Bond group (*P*=0.999). Significantly higher bond strength values were observed at RMGI group as the Biodentine aging period increased (*P*<0.02).

## Discussion

Biodentine is a calcium silicate based material with improved mechanical properties [17, 18] and with good biocompatibility [19, 20] as well as a bioactive behavior [21]. Unlike MTA which has an extended setting period [22, 23], Biodentine is a fast-setting calcium-silicate cement (ranging from 6.5 min to 45 min) [23]. This is achieved by increasing particle size and adding calcium chloride as a water reducing agent [13, 23]. Its compressive strength is higher than MTA which is attributed to the low water/powder ratio [13, 23]. Torabinejad *et al*. [22] observed an increase in compressive strength of MTA to 67 MPa after 21 days. Different compressive strength values have been reported for Biodentine after one day storage ranging between 61.35 MPa [24] to 78.5 MPa [25], which might indicate immediate application of overlying materials over Biodentine. Considering above, Biodentine has been recommended for use as a pulp capping agent [19] and dentin substitute under restorations [13, 26]. The bond strength between restorative materials and Biodentine at various application periods is important for the quality of restorations and their longevity [9, 16].

In this study, the bond strength of a resin composite bonded using three different adhesive systems and a RM-GIC to aged Biodentine specimens overtime were evaluated. The bond strength test was delayed for two days after the application of resinous materials since the polymerization of resin materials continues after irradiation over time [27, 28] which affect the bond strength [29, 30]. In the current study, the bonds strength varied among different aging periods and adhesive materials. So, the null hypothesis was rejected. The mean bond strength values ranged from 2.72±0.62 to 62.49±16.39 MPa with the lowest values being obtained for the control group, which received no adhesive agent, and the highest with the All-Bond Universal adhesive applied over freshly mixed Biodentine. However, the bond strength of the All-Bond Universal decreased significantly for specimens incubated for a longer period of time. The low viscosity of All-Bond Universal may make it penetrate into the porosities [31, 32] of recently set Biodentine cement [33]. In addition, this adhesive has 10-methacryloyloxydecyl dihydrogen phosphate (MDP) in its composition [31, 34] which could be effective in achieving a chemical interaction [35] with the calcium rich [3, 36] Biodentine surface. The assumed micro-mechanical and chemical interaction of All-Bond Universal and Biodentine might explain the high bond strength of All-Bond Universal to Biodentine at 12 min. However, this adhesive agent was associated with lower bond strengths to aged Biodentine specimens at one week and one month, which might be explained by the increased surface hardness of Biodentine over time [37] which reduces the penetration of this ultra-mild acidic adhesive agent (pH>3) [31, 34] resulting in a shallower etching pattern and reduced micro-mechanical retention.

The bond strength values of all other adhesives to Biodentine blocks incubated for one week were significantly higher than the micro-shear bond strength measure at 12 min. The hydration process of calcium silicate cements undergoes several stages and the resulting hydrate cement in its early setting stages is poorly crystallized and highly porous [3]. Adhesive agent application and the curing contraction of resin composite might stress the porous un-matured Biodentine cement [16] at this early setting stage to adversely affect the bond strength. This demonstrates the importance of allowing Biodentine to mature for a longer period of time prior to the application of the adhesive materials.

The results of this study also revealed that the bond strength values of the two-step self-etch adhesive and All-Bond Universal to one month aged Biodentine specimens were lower than those obtained after one week. This might be explained by the fact that the setting reaction continues over time and the porous calcium silicate hydrate hardens to form a solid network with improved crystallinity and decreased porosity [3, 36], and increased hardness that could affect its interfacial behavior with mild or ultra-mild self-etch adhesive systems. However, there was no significant difference in bond strength values of etch and rinse adhesive system to one week and one month incubated Biodentine blocks. This might imply that Biodentine matured sufficiently after one week to cause similar etching patterns and irregularities by phosphoric acid agent as occurred at one month.

Clearfil SE Bond had lower bond strength to recently set Biodentine in comparison to All-Bond Universal. The more aggressive acidic primer (pH≈2) [38] of this bonding agent in comparison to All-Bond Universal might adversely affect Biodentine superficial gel-like amorphous structure [10], calcium silicate hydrate gel (CSH gel) [3], and the newly formed crystalline structure [3, 36], which could impede further hydration of the cement grains at the superficial layers. Consequently, the bond strength to the damaged Biodentine surface is likely to be reduced.

In this study, etch and rinse adhesive system (Adper Single Bond 2) had higher micro-shear bond strength values on one week aged Biodentine specimens in comparison to the two-step self-etch adhesive (Clearfil SE Bond). It can be concluded that the phosphoric acid agent may create more pronounced and retentive porosities on Biodentine surface into which the adhesive can penetrate compared to the dissolution depth obtained with Clearfil SE Bond.

**Table 2:** Mean micro-shear bond strength and standard deviation values of adhesive systems (MPa) to aged Biodentine^TM^

**Adhesive system**	**Aging period**		
	**12 min**	**1 week**	**1 month**
**No Adhesive **	2.76±0.62	8.12±2.29	3.15±1.29
**Adper** ^TM^ ** Single Bond **	9.26±2.66	25.41±2.55	25.02±8.93
**Clearfil SE Bond **	5.72±3.23	18.52±1.82	15.69±1.23
**All-Bond Universal **	62.49±16.39	31.29±3.94	19.59±4.38
**Fuji II LC GIC**	6.21±3.19	15.92±3.51	28.59±9.68

The bond strength of RM-GIC increased over time probably due to the improved chemical reaction between hydrated calcium silicate gel (CSH gel) and hydration products [36] such as calcite [37] and the carboxyl group of poly acids in GIC. The increased micro-shear bond strength values of RM-GIC over aged Biodentine specimens might imply that a more stable and constant result could be achieved by layering RM-GIC over Biodentine. In addition, RM-GIC might not have the adverse effect of adhesive materials on the surface properties of Biodentine. 

Several studies have evaluated the bond strength of restorative materials to Biodentine with adhesive systems for the purpose of outcome comparison. Odabas *et al*. [14] evaluated the shear bond strength of three different adhesive systems (etch-and-rinse adhesive, two-step self-etch adhesive and one-step self-etch adhesive systems) to Biodentine stored for 12 min and 24 h. No significant differences were found between all of the adhesive groups at the same time intervals (12 min and 24 h). Between the two time intervals, the lowest value was obtained for the etch-and-rinse adhesive at the 12-min period, and the highest was obtained for the two-step self-etch adhesive at 24-h. The placement of composite resin used with self-etch adhesive systems over Biodentine showed better shear bond strength values [14]. Likewise, the current study did not show significant different in micro-shear bond strength values between two step self-etch and etch and rinse adhesive systems to similarly aged Biodentine specimens (*P*≥0.19) except to one-week aged substrate (*P*˂0.001) which the latter adhesive agent had higher micro-shear bond strength values than the former.

In another study, *in vitro* micro-shear bond strengths of a resin composite to Biodentine versus a glass ionomer or a resin modified glass ionomer cement using a universal adhesive agent in self-etch and total-etch mode after aging were compared [16]. Biodentine was a weak restorative material in its early setting phase. Thus, placing the overlying resin composite as part of the layered definitive restoration was best delayed for more than two weeks to allow sufficient intrinsic maturation to withstand contraction forces from the resin composite [16]. No significant difference was found between the self-etch and total etch adhesive modes [16]. The observation of the current study is in agreement with the finding of Hashem DF *et al*. [16] study for delayed application of resinous materials, except for All-Bond Universal in self-etch mode which showed the highest bond strength values to Biodentine at 12 min aging period. This may allow immediate application of resin composite over freshly mixed Biodentine using All-Bond Universal. Obviously, other types of calcium silicates cements with similar setting characteristics should be evaluated to achieve more reliable result. Further investigations to better understand the adhesion mechanism of adhesive systems to Biodentine and the structural alterations during the adhesive procedure are recommended.

## Conclusion

Within the limitation of this study, it was concluded that there is a difference in micro-shear bond strength of various adhesives bonded to different aged Biodentine blocks. Universal bonding agent in self-etch mode applied over Biodentine specimens at 12-min setting time exhibited significantly higher mean micro-shear bond strength compared to other groups. Also, it was found that if a longer waiting time can be scheduled after mixing of Biodentine, higher shear bond strength measurements values can be expected with all other adhesives.
